# Intravesical migration of an intrauterine device

**DOI:** 10.1186/s13104-015-1792-6

**Published:** 2016-01-02

**Authors:** Christian Kofi Gyasi-Sarpong, Patrick Opoku Manu Maison, Emmanuel Morhe, Ken Aboah, Kwaku Addai-Arhin Appiah, Roland Azorliade, Kofi Baah-Nyamekye, Kwaku Otu-Boateng, George Amoah, Isaac Antwi, Benjamin Frimpong-Twumasi, Douglas Arthur

**Affiliations:** Department of Surgery, School of Medical Sciences, KNUST, Kumasi, Ghana; Department of Surgery, Komfo Anokye Teaching Hospital, Kumasi, Ghana; Department of Obstetrics and Gyneacology, School of Medical Sciences, KNUST, Kumasi, Ghana

**Keywords:** Intrauterine contraceptive device, Intravesical migration, UTI

## Abstract

**Background:**

Intrauterine contraceptive device is the most common method of reversible contraception in women. The intrauterine contraceptive device can perforate the uterus and can also migrate into pelvic or abdominal organs. Perforation of the urinary bladder by an intrauterine contraceptive device is not common. In West Africa, intravesical migration of an intrauterine contraceptive device has been rarely reported. In this report, we present a case of an intrauterine contraceptive device migration into the urinary bladder of a 33 year old African woman at the Komfo Anokye Teaching Hospital, Kumasi, Ghana.

**Case report:**

A 33 year old African woman presented with persistent urinary tract infection of 7 months duration despite appropriate antibiotic treatments. An abdominal ultrasonography revealed a urinary bladder calculus which was found to be an intrauterine contraceptive device on removal at cystoscopy. She got pregnant whilst having the intrauterine contraceptive device in place and delivered at term.

**Conclusion:**

The presence of recurrent or persistent urinary tract infection in any woman with an intrauterine contraceptive device should raise the suspicion of intravesical migration of the intrauterine contraceptive device.

## Background

Intrauterine contraceptive device (IUCD) is the most common method for reversible contraception in women because it is effective, safe, and cost effective [1]. One of the major complications of IUCD is perforation of the uterus and the migration of the device into pelvic or abdominal organs [2]. However, intravesical migration of IUCD is not common. IUCD insertion is routinely performed in Komfo Anokye Teaching Hospital (KATH), the second largest teaching hospital in Ghana. There has been no report of intravesical migration of IUCD in Ghana and same has rarely been reported in the West African sub-region. In Nigeria, Eke et al. reported a case of intravesical migration of IUCD [3].

We present the case of a 33-year-old African woman with persistent *Escherichia coli* (*E. coli*) urinary tract infection (UTI) due to intravesical migration of an IUCD appearing as a bladder calculus on ultrasonography.

## Case presentation

A 33-year-old African woman, para 4 with one spontaneous abortion was referred to the Urology clinic with complaints of dysuria, strangury, frequency of micturition, urgency, nocturia and severe lower abdominal pain. These symptoms had persisted for 7 months despite repeated treatment for *E. coli* isolated urinary tract infections by her attending gynaecologist. She used an IUCD for contraception for 2 years after the birth of her second child. The IUCD was removed before her third pregnancy. Forty (40) days after her third delivery, a copper T IUCD was inserted by her gynaecologist.

The patient reported after 21 months post IUCD insertion with symptoms of pregnancy. Further evaluation revealed she was 5 months pregnant. The IUCD strings were not visible on gynaecologic examination and transvaginal ultrasonography by the gynaecologist failed to detect the IUCD. Thus, the IUCD was assumed to have fallen out, permitting the patient to have her fourth pregnancy. She had an uneventful pregnancy and was delivered by a lower uterine segment caesarean section at term on account of two previous caesarean sections.

**Fig. 1:**
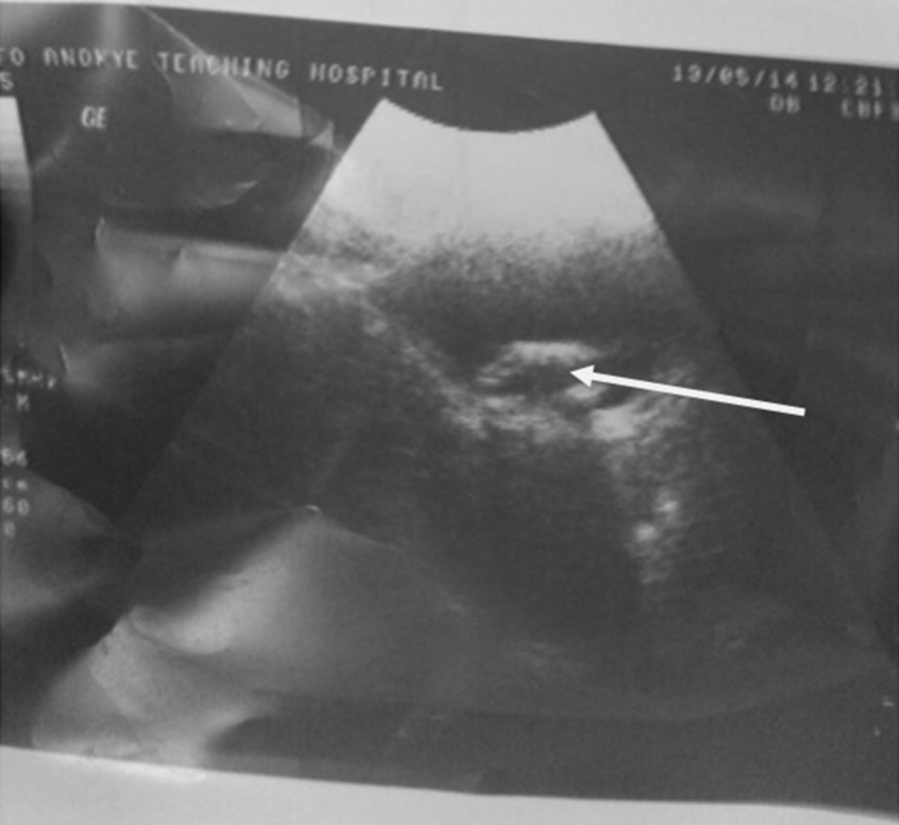
Abdominal ultrasonography showing the intrauterine contraceptive device as a bladder calculus (*arrowed*)

She started experiencing lower urinary tract symptoms 15 months post delivery. Urine culture persistently isolated *E. coli* despite appropriate antibiotic treatments by the attending gynaecologist. She was therefore referred to the urologist for further evaluation and management. An abdominal ultrasonography by the radiologist revealed a urinary bladder calculus (Fig. 1). This turned out to be a copper T IUCD at cystoscopic removal. The patient’s urinary tract symptoms resolved completely after the removal of the IUCD.

## Discussion

Intrauterine contraceptive device (IUCD) is the most common method for reversible contraception in women because it is safe, inexpensive and readily available [1]. Uterine perforation and migration of the IUCD into abdominal or pelvic organs is a major complication of IUCD insertion [2] with an incidence of 1.9–3.6 per 1000 insertions [4]. Factors influencing the risk of perforation include the type of IUCD used, the time of insertion, the insertion technique, and anatomy of the cervix and uterus [4]. The exact mode of uterine perforation and IUCD migration is unclear [5]. However, it is believed that perforation mostly occurs at the time of insertion but may also occur spontaneously at a later time or during puerperium [6].

The presence of pain and bleeding per vaginam after IUCD insertion suggests that uterine perforation may have occurred at the time of insertion [7]. Secondary perforation may be due to slow migration through the myometrium which may be enhanced by spontaneous uterine contractions [8]. When a pregnancy occurs in a patient with an IUCD, there must be a high suspicion of uterine perforation and possible migration [1]. IUCDs which migrate to the urinary bladder are either located in the bladder wall or within the bladder lumen [1].

Most patients with intravesical migration of IUCD are symptomatic [8] with UTI being the most common presentation [9]. The patient in this case presented with persistent UTI.

Transvaginal ultrasonography is the investigation of choice for locating the intravesical IUCD [8]. However, in this case a transabdominal ultrasonography by the radiologist showed the intravesical IUCD as a bladder calculus. Ultrasonography is operator-dependent and this may have accounted for the failure to detect the IUCD by the gynaecologist.

Cystoscopy is another means of visualising the intravesical IUCD and is helpful for its removal [8].

All IUCDs which have migrated into the urinary bladder must be removed even if they are asymptomatic. This is to prevent complications such as calculus formation and bladder rupture [1].

An IUCD which has migrated into the urinary bladder is treated by cystoscopic removal or by open suprapubic cystotomy [10].

Cystoscopic removal is preferred because it has a low morbidity and is highly effective [11]. In this case, cystoscopic removal was done successfully. Open surgery is currently restricted to centres without cystoscopic facilities and also for the removal of IUCDs with calculus formation that are not amenable to cystoscopic removal [7]. Laparoscopic removal, a minimally invasive alternative to open surgery can also be used [5].

## Conclusion

The presence of recurrent or persistent UTI in any woman who gets pregnant despite having had an IUCD inserted should raise the suspicion of intravesical migration of the IUCD. Early ultrasonography for the cause of recurrent or persistent UTI is recommended.

## Abbreviations

IUCD: intrauterine contraceptive device
KATH: Komfo Anokye Teaching Hospital
UTI: urinary tract infection
*E. coli*: *Escherichia coli*
